# Association of gravidity, parity, and abortion history with nodal metastasis in women with papillary thyroid carcinoma: a retrospective cohort study

**DOI:** 10.3389/fendo.2026.1856799

**Published:** 2026-08-06

**Authors:** Kai Wang, Hanyu Wang, Dongqiang Yang, Yanzhao Wu, Lan Zhang, Yan Liu, Ping Shi

**Affiliations:** 1 Department of Head and Neck Surgery, National Cancer Center/National Clinical Research Center for Cancer/Cancer Hospital, Chinese Academy of Medical Sciences and Peking Union Medical College, Beijing, China; 2 Department of Otolaryngology Head and Neck Surgery, The Fourth Hospital of Hebei Medical University and Hebei Tumor Hospital, Shijiazhuang, China; 3 Department of Radiological Intervention, The Fourth Hospital of Hebei Medical University and Hebei Tumor Hospital, Shijiazhuang, China

**Keywords:** central lymph node metastasis, gravidity, lymph node metastasis, papillary thyroid carcinoma, reproductive history

## Abstract

**Background:**

Papillary thyroid carcinoma (PTC) predominantly affects women, particularly during the reproductive years, suggesting that reproductive factors may influence not only tumor occurrence but also metastatic behavior. However, evidence regarding the association between reproductive history and lymph node metastasis (LNM) in women with established PTC remains limited.

**Methods:**

We conducted a retrospective cohort study of 1,828 women with pathologically confirmed PTC who underwent initial surgery. The primary outcome was any cervical lymph node metastasis (LNM). Secondary outcomes included isolated central lymph node metastasis (CLNM) and combined central and lateral lymph node metastasis (CLNM+LLNM). Multivariable logistic regression models were used to evaluate the associations between reproductive history, gravidity, parity, and abortion history and nodal outcomes. Reproductive variables were analyzed in separate models to reduce collinearity. Sensitivity and subgroup analyses were performed to examine the stability of the observed associations.

**Results:**

Cervical LNM was identified in 903 patients (49.4%). In the primary adjusted model, ever pregnancy was associated with lower odds of any LNM (adjusted OR 0.420, 95% CI 0.276-0.640; *P<0.001*). Compared with nulligravid women, the adjusted ORs for any LNM were 0.404 (95% CI 0.260-0.628), 0.480 (95% CI 0.306-0.751), and 0.356 (95% CI 0.224-0.567) for women with 1, 2, and ≥3 pregnancies, respectively (*P* for trend <0.001). Similar inverse associations were observed for isolated CLNM and CLNM+LLNM. However, the category-specific estimates were not strictly monotonic, suggesting a threshold-like rather than a linear dose-response pattern. Similar inverse associations were observed for isolated CLNM and CLNM+LLNM in the primary models. In sensitivity analyses, the associations generally remained directionally inverse but were attenuated in some analyses, including models using flexible adjustment for continuous age. Analyses involving lateral disease were interpreted cautiously because lateral neck dissection was not performed uniformly across the cohort.

**Conclusions:**

In women with PTC, ever pregnancy was associated with lower odds of cervical LNM, particularly for central compartment disease. This association appeared to be more closely related to ever having been pregnant than to a linear increase in gravidity or parity. Reproductive history may provide supplementary context for nodal risk assessment, but it should not replace established clinicopathological factors or independently alter surgical decision-making.

## Introduction

Thyroid cancer is one of the most common endocrine malignancies, and its incidence has risen substantially over recent decades, particularly in women (1–4). Papillary thyroid carcinoma (PTC) is the predominant histological subtype. Although disease - specific survival is generally excellent, cervical lymph node metastasis (LNM) remains clinically important because it is associated with locoregional recurrence, reoperation, and a greater treatment burden (5–9). In routine surgical practice, decisions regarding the extent of thyroidectomy and cervical lymph node dissection are mainly guided by imaging findings and established tumor - related factors, including tumor size, multifocality, bilaterality, and extrathyroidal extension (ETE) (5, 9).

Even so, the biological heterogeneity of PTC suggests that host-related factors may also contribute to metastatic behavior. One of the most consistent epidemiological features of thyroid cancer is its marked female predominance, particularly during the reproductive years (1, 2). This pattern has long piqued interest in the possible role of reproductive and hormonal exposures in thyroid tumorigenesis. Most previous studies, however, have focused on cancer occurrence rather than the metastatic phenotype after diagnosis. Meta - analyses and epidemiologic studies have suggested that some menstrual and reproductive variables may be related to thyroid cancer risk, although the evidence remains heterogeneous (10–13). In our previous work, female sex was associated with a lower likelihood of LNM in PTC, and among women, reproductive factors were also linked to nodal risk (14). These observations suggest that reproductive history may be relevant not only to disease susceptibility but also to the pattern and extent of nodal spread once PTC has developed.

From a surgical standpoint, cervical nodal metastasis in PTC should be interpreted in a compartment-specific manner rather than as a single uniform endpoint. Central lymph node metastasis is usually the earliest and most common pattern of regional spread, whereas lateral nodal disease often reflects a broader extent of dissemination and has more direct implications for operative planning. In everyday practice, however, lateral neck dissection is generally reserved for patients with clinically suspected or pathologically confirmed lateral disease. As a result, lateral nodal involvement is more vulnerable to occult disease, underdetection, and selection-related bias than central compartment metastasis. This distinction is also consistent with our group’s subsequent work on lateral neck disease, which has highlighted the need for compartment-oriented risk assessment in PTC rather than treating all nodal metastases as interchangeable (15–18).

Against this background, we conducted a retrospective cohort study of 1,828 women with PTC to investigate whether reproductive history, gravidity, parity, and abortion history were associated with pathological cervical nodal metastasis. To better reflect the clinical and biological heterogeneity of nodal spread, we evaluated any LNM, isolated central lymph node metastasis, and lateral nodal involvement as separate outcomes. We also performed prespecified sensitivity analyses and prediction analyses to assess the robustness and potential clinical relevance of the findings.

## Materials and methods

### Study design and population

This retrospective cohort study included adult women who underwent initial surgery for PTC at the Department of Otorhinolaryngology Head and Neck Surgery, Western Campus of the Fourth Hospital of Hebei Medical University, between January 2015 and December 2019. All diagnoses were confirmed by postoperative pathology. Eligible patients met all of the following criteria: female sex, age ≥18 years, initial thyroid surgery for pathologically confirmed PTC, thyroidectomy with central neck dissection with or without lateral neck dissection, and complete clinicopathological data for the planned analyses. Patients were excluded if they had a non-PTC pathological diagnosis, previous thyroid or neck surgery, unclear primary tumor status, or severe systemic disease that materially affected perioperative management. Women with missing reproductive-history information were excluded before construction of the final analytic cohort; therefore, all women included in the analysis had available reproductive-history data. The study was approved by the Ethics Committee of the Fourth Hospital of Hebei Medical University and Hebei Tumor Hospital (approval No. 2024KS009) and was conducted in accordance with the Declaration of Helsinki.

### Variables and definitions

Clinical variables included age at diagnosis, body mass index (BMI), hypertension, type 2 diabetes mellitus, history of malignancy, thyroid-stimulating hormone (TSH), and reproductive history. Pathological variables included tumor size, multifocality, bilaterality, extrathyroidal extension, Hashimoto’s thyroiditis, and pathological nodal status. Reproductive history was defined as at least one pregnancy(included any pregnancy outcomes, such as ectopic pregnancy, miscarriage, and live birth) before thyroid surgery. Gravidity and parity were categorized to allow dose-response analyses. Abortion history was categorized as no gravidity, gravidity without abortion, one abortion, and two or more abortions, as recorded in the medical record.

### Outcomes

Pathological nodal status was classified into four mutually exclusive categories: no lymph node metastasis (No LNM), isolated central lymph node metastasis (isolated CLNM), isolated lateral lymph node metastasis (isolated LLNM), and combined central and lateral lymph node metastasis (CLNM+LLNM). For clinically oriented analyses, we additionally defined two derived categories (1): any LNM, indicating the presence of any pathological cervical nodal metastasis; and (2) lateral involvement, defined as pathological lateral nodal disease of any type, including isolated LLNM and CLNM+LLNM. The primary outcome was any cervical lymph node metastasis (LNM). Secondary outcomes included isolated central lymph node metastasis (CLNM) and combined central and lateral lymph node metastasis (CLNM+LLNM). Because lateral neck dissection was not performed uniformly across the entire cohort, analyses involving lateral disease were interpreted cautiously.

### Statistical analysis

Continuous variables are reported as mean ± standard deviation, and categorical variables as number (percentage). Between-group comparisons were performed using Student’s t test or the Mann-Whitney U test for continuous variables and the chi-square test or Fisher’s exact test for categorical variables, as appropriate. All statistical tests were two-sided, and P<0.05 was considered statistically significant.

Binary logistic regression was used to evaluate factors associated with any LNM. Additional multivariable logistic regression models were fitted for isolated CLNM and for lateral involvement and CLNM+LLNM, using No LNM as the reference category. Candidate covariates were selected *a priori* on the basis of clinical relevance and previous literature and included age, BMI, hypertension, type 2 diabetes mellitus, history of malignancy, Hashimoto’s thyroiditis, TSH, tumor size, multifocality, bilaterality, and extrathyroidal extension. In the primary models, age was coded as a binary variable using 45 years as the cutoff (age ≥45 years vs <45 years).

Reproductive history was defined as ever pregnancy, corresponding to gravidity ≥1. Nulligravid women were used as the reference group for analyses of ever pregnancy and gravidity categories. Because the binary reproductive-history analysis and the gravidity-category analysis shared the same nulligravid reference group, the gravidity-category analysis was interpreted as a category-specific decomposition of the ever-pregnancy contrast rather than as an independent line of evidence. Because reproductive history, gravidity, parity, and abortion history were highly correlated, these variables were entered into separate multivariable models to reduce collinearity. For abortion-history analyses, an additional analysis restricted to ever-gravid women was performed, using women with gravidity but no abortion history as the reference group.

For the main multivariable analyses, candidate covariates were first entered into forward likelihood-ratio logistic regression procedures to identify variables retained in the final outcome-specific models. To reduce the possibility of selective reporting, complete multivariable models including all candidate covariates were additionally presented in the **Supplementary Materials**.

Sensitivity analyses included crude and adjusted logistic regression, an alternative adjustment model, restriction to tumors ≥1 cm, a restricted cohort with pathological lateral neck evaluation, and 1:1 propensity score matching (PSM) followed by conditional logistic regression. To address potential residual confounding due to non-linear age effects, the primary models were repeated by replacing the binary age term with restricted cubic spline terms for continuous age. Restricted cubic spline terms were generated using four knots placed at the 5th, 35th, 65th, and 95th percentiles of the age distribution. The remaining covariates were kept consistent with the primary adjusted models. Age distribution was also summarized across gravidity categories, and age-stratified analyses were performed using predefined age strata.

A baseline clinicopathological prediction model for lymph node metastasis was constructed using predefined covariates. To evaluate the incremental predictive value of reproductive factors, reproductive history and gravidity were added separately to the baseline model. Model performance was assessed in terms of discrimination, calibration, and clinical usefulness using decision curve analysis. For isolated CLNM, a restricted dataset including only patients with isolated CLNM and those without lymph node metastasis was used to construct an outcome-specific prediction model.

## Results

### Baseline characteristics

A total of 1,828 women with papillary thyroid carcinoma were included in the analysis. The mean age at diagnosis was 45.53 ± 11.80 years, and the mean tumor size was 1.13 ± 0.84 cm. 778 patients (42.6%) had a body mass index >25 kg/m², 318 (17.4%) had hypertension, 103 (5.6%) had type 2 diabetes mellitus, 58 (3.2%) had a history of malignancy, and 149 (8.2%) had Hashimoto’s thyroiditis. Multifocal tumors were present in 621 patients (34.0%), bilateral disease in 424 (23.2%), and extrathyroidal extension (ETE) in 385 (21.1%). Reproductive history was documented in 1,691 women (92.5%),who had ever been pregnant; the remaining 137 were nulligravid.

Overall, 903 patients (49.4%) had pathological cervical lymph node metastasis (LNM). Among these, 572 (31.3%) had isolated central lymph node metastasis (CLNM), 52 (2.9%) had isolated lateral lymph node metastasis (LLNM), 279(15.3%)combined central and lateral lymph node metastasis (CLNM+LLNM) and 331(18.1%) had lateral nodal involvement. The baseline clinicopathological characteristics of the cohort are summarized in **Table 1**.

**Table 1:** Baseline clinicopathological characteristics of the study cohort.

Variable	Overall cohort (n = 1,828)(%)
Age, years, mean ± SD	45.53 ± 11.80
BMI >25 kg/m², n (%)	778 (42.6)
Hypertension, n (%)	318 (17.4)
Type 2 diabetes mellitus, n (%)	103 (5.6)
History of malignancy, n (%)	58 (3.2)
Hashimoto’s thyroiditis, n (%)	149 (8.2)
TSH, mIU/L, mean ± SD	2.26 ± 1.92
Tumor size, cm, mean ± SD	1.13 ± 0.84
Tumor size >1 cm, n (%)	648 (35.4)
Multifocality, n (%)	621 (34.0)
Bilaterality, n (%)	424 (23.2)
Extrathyroidal extension, n (%)	385 (21.1)
Reproductive history, n (%)	1,691 (92.5)
Nulligravidity, n (%)	137(7.5)
Pathological nodal status	
Nodal pattern	n (%)
No LNM	925 (50.6)
Isolated CLNM	572 (31.3)
Isolated LLNM	52 (2.9)
Lateral involvement	331(18.1)
CLNM+LLNM	279(15.3)

Baseline clinicopathological characteristics of the study cohort. Data are presented as mean ± standard deviation or n (%), unless otherwise indicated. Pathological nodal patterns were classified as no lymph node metastasis (No LNM), isolated central lymph node metastasis (isolated CLNM), isolated lateral lymph node metastasis (isolated LLNM), and combined central and lateral lymph node metastasis (CLNM+LLNM). For derived analyses, lateral involvement was defined as any pathological lateral nodal metastasis, including isolated LLNM and CLNM+LLNM.

### Association of reproductive history with any lymph node metastasis

In univariable analysis, reproductive history was associated with a lower likelihood of any LNM (OR 0.335, 95% CI 0.227-0.495; *P* < 0.001). This association remained significant after adjustment for clinically relevant covariates (adjusted OR 0.420 95% CI 0.276-0.640; *P* < 0.001) (**Table 2**). Among conventional clinicopathological factors, tumor size >1 cm, bilaterality, and extrathyroidal extension were independently associated with increased nodal metastatic risk. In contrast, BMI, hypertension, history of malignancy, Hashimoto’s thyroiditis, and TSH were not significantly associated with LNM in the adjusted model.

**Table 2:** Univariable and multivariable logistic regression analyses for any cervical lymph node metastasis in women with PTC.

Variable	Univariable OR 95% CI	*P* value	Multivariable OR 95% CI	*P* value
Reproductive history (yes vs no)	0.335 0.227-0.495	<0.001^a^	0.420 0.276-0.640	<0.001^a^
Age, years(<45 vs ≥45)	0.479 0.397-0.577	<0.001^a^	0.460 0.372-0.568	<0.001^a^
BMI >25 kg/m²	0.920 0.764-1.107	0.378		
Hypertension	0.898 0.705-1.144	0.382		
Type 2 diabetes mellitus	0.634 0.422-0.953	0.028^a^		
History of malignancy	0.716 0.421-1.217	0.216		
Hashimoto’s thyroiditis	0.927 0.663-1.296	0.656		
TSH, mIU/L	1.024 0.847-1.239	0.803		
Tumor size >1 cm	3.608 2.942-4.425	<0.001^a^	3.021 2.431-3.756	<0.001^a^
Multifocality	1.721 1.415-2.093	<0.001^a^		
Bilaterality	2.054 1.642-2.568	<0.001^a^	1.940 1.522-2.472	<0.001^a^
Extrathyroidal extension	2.372 1.875-3.000	<0.001^a^	1.902 1.465-2.469	<0.001^a^

^a^ Statistically significant (*P*<0.05). All statistical tests were two-sided. The outcome variable was any pathological cervical lymph node metastasis. Odds ratios and 95% confidence intervals were estimated using binary logistic regression. The primary multivariable model was adjusted for age, BMI, hypertension, type 2 diabetes mellitus, Reproductive history, Hashimoto’s thyroiditis, TSH, tumor size, multifocality, bilaterality, and extrathyroidal extension. OR, odds ratio; CI, confidence interval; BMI, body mass index; TSH, thyroid-stimulating hormone. No adjustment for multiple comparisons across numerous models/outcomes.

### Pattern-specific analyses of nodal metastasis

To further explore compartment-specific patterns, separate multivariable models were constructed for isolated CLNM, lateral nodal involvement and CLNM+LLNM(**Table 3**). **Table 3** presents the final forward-selected models. Reproductive history remained inversely associated with isolated CLNM (adjusted OR 0.413, 95% CI 0.266-0.642; *P* < 0.001), lateral nodal involvement (adjusted OR 0.414, 95% CI 0.229-0.749; *P* = 0.004), and CLNM+LLNM (adjusted OR 0.345, 95% CI 0.186-0.641; *P* = 0.001). Tumor size >1 cm was consistently associated with increased odds of nodalmetastasis across outcome models, with the strongest association observed for CLNM+LLNM (OR 5.875, 95% CI 4.256-8.111; *P* < 0.001). Bilaterality and extrathyroidal extension were also associated with increased nodal risk in several outcome-specific models. Complete fully adjusted estimates for all candidate covariates, including variables not retained in the forward-selected models, are provided in **Supplementary Table 1**.

**Table 3:** Separate multivariable logistic regression analyses for pathological nodal metastatic patterns in women with papillary thyroid carcinoma.

Variable	Isolated CLNM vs No LNM OR 95% CI	*P* value	Isolated LLNM vs No LNM OR 95% CI	*P* value	Lateral involvement vs No LNM OR 95% CI	*P* value	CLNM+LLNM vs No LNM OR 95% CI	*P* value
Reproductive history (yes vs no)	0.413 0.266-0.642	<0.001^a^			0.414 0.229-0.749	0.004^a^	0.345 0.186-0.641	0.001^a^
Age, years (<45 vs ≥45)	0.441 0.349-0.556	<0.001^a^			0.496 0.364-0.675	<0.001^a^	0.427 0.304-0.599	<0.001^a^
Hashimoto’s thyroiditis			2.514 1.164-5.430	0.019^a^				
Tumor size >1 cm	2.400 1.883-3.060	<0.001^a^	2.053 1.120-3.762	0.020^a^	4.735 3.529-6.353	<0.001^a^	5.875 4.256-8.111	<0.001^a^
Multifocality					1.810 1.145-2.861	0.011^a^	1.819 1.089-3.039	0.022^a^
Bilaterality	1.509 1.144-1.989	0.004^a^			1.765 1.078-2.881	0.024^a^	2.152 1.251-3.702	0.006^a^
Extrathyroidal extension	1.603 1.194-2.151	0.002^a^	2.311 1.203-4.440	0.012^a^	2.581 1.842-3.616	<0.001^a^	2.766 1.923-3.978	<0.001^a^

^a^ Statistically significant (*P*<0.05). All statistical tests were two-sided. The reference category was No LNM. Adjusted odds ratios and 95% confidence intervals were estimated for isolated central lymph node metastasis, isolated lateral lymph node metastasis, and lateral involvement. Because isolated lateral nodal events were sparse, lateral involvement was additionally analyzed as a derived endpoint that combined isolated LLNM and CLNM+LLNM. Models values are variables retained in the final multivariable logistic regression models selected using forward likelihood-ratio procedures. All candidate covariates were entered into the model selection procedure, including age, BMI, hypertension, type 2 diabetes mellitus, history of malignancy, Hashimoto’s thyroiditis, TSH, tumor size, multifocality, bilaterality, extrathyroidal extension, and reproductive-history variables. Blank cells indicate that the corresponding variable was not retained in the final selected model for that outcome, rather than that the variable was not considered. Complete multivariable estimates for all candidate covariates in each outcome model, including non-significant estimates, are provided in **Supplementary Table 1**. aOR, adjusted odds ratio; CI, confidence interval; LNM, lymph node metastasis; CLNM, central lymph node metastasis; LLNM, lateral lymph node metastasis. No adjustment for multiple comparisons across numerous models/outcomes.

### Subgroup analyses

**Figure 1:**
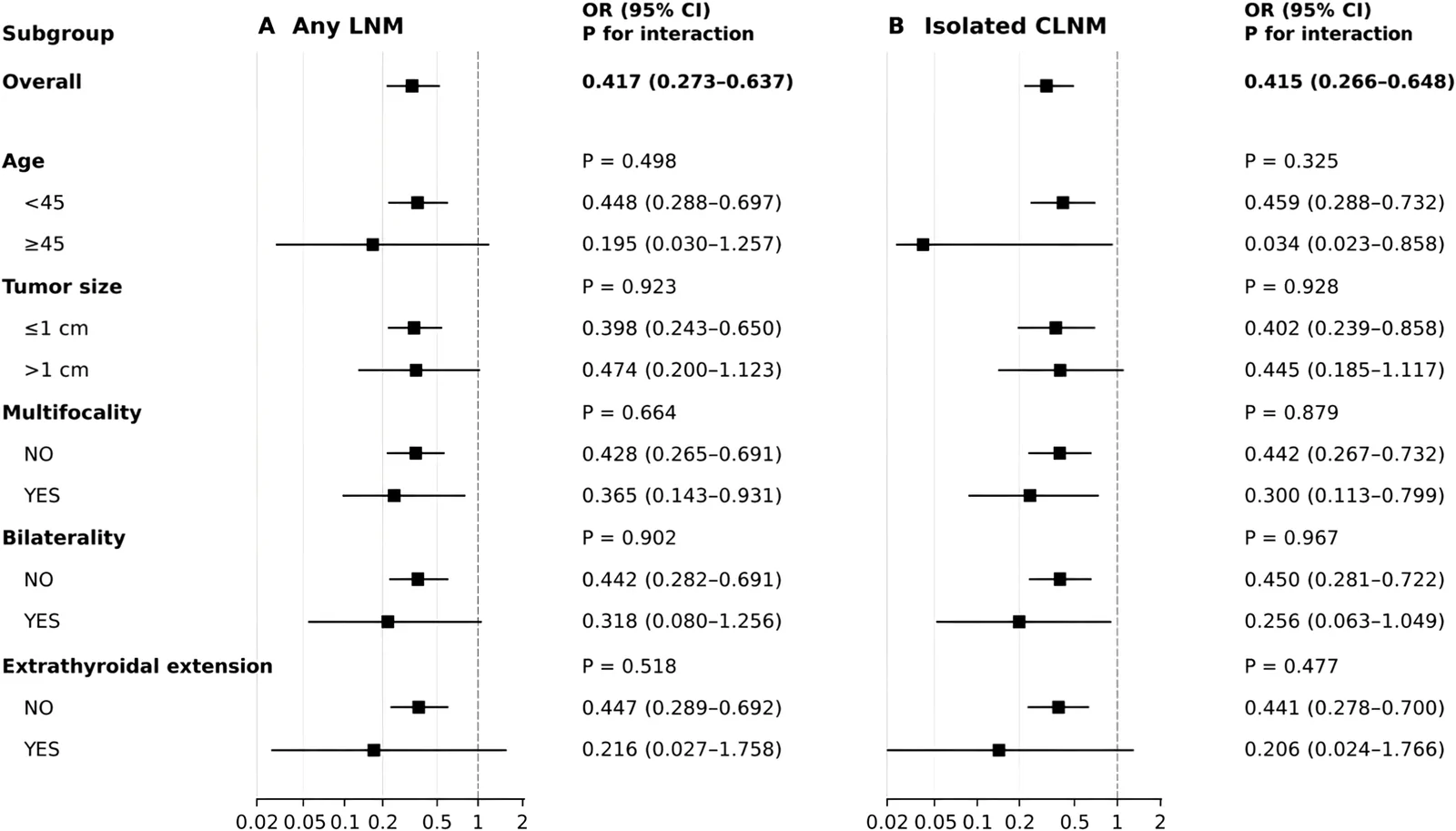
Subgroup analyses of the association between reproductive history and nodal metastasis. Forest plots showing subgroup-specific adjusted odds ratios and 95% confidence intervals for any lymph node metastasis and isolated central lymph node metastasis. The association between reproductive history and nodal metastasis was generally directionally inverse across major clinicopathological subgroups.

Subgroup analyses showed that the association between reproductive history and nodal metastasis was generally directionally inverse across major clinicopathological strata (**Figure 1**). The direction of the estimate was largely similar in subgroups defined by tumor size, bilaterality, and extrathyroidal extension. However, subgroup - specific estimates for lateral nodal disease were more variable, and several strata could not be estimated due to sparse events. Therefore, subgroup analyses were interpreted as exploratory and were not used as definitive evidence of effect consistency.

### Sensitivity analyses

Sensitivity analyses generally supported the direction of the primary association, although several estimates were attenuated and approached the threshold for statistical significance (**Table 4**). The inverse association between reproductive history and any LNM was observed in crude and adjusted models and in the alternative adjustment model. Similar directionally inverse estimates were observed for isolated CLNM and CLNM+LLNM in outcome-specific models. In the cohort restricted to patients with tumors ≥1.0 cm, the association remained statistically significant but was close to the significance boundary (OR 0.534, 95% CI 0.288-0.990; *P* = 0.046). In the 1:1 propensity score-matched analysis, reproductive history remained associated with lower odds of any LNM (OR 0.516, 95% CI 0.289-0.922; *P* = 0.026), although the matched analysis was based on a substantially reduced sample size. The association between reproductive history and isolated CLNM was attenuated after matching and did not reach statistical significance. Although the inverse association between reproductive history and LNM remained directionally similar in age-stratified analyses, the estimates were attenuated and were not statistically significant in some age strata (**Supplementary Tables 2**, **3**). When continuous age was modeled flexibly using restricted cubic splines, the associations were further attenuated. The estimate remained directionally inverse for any LNM but no longer reached conventional statistical significance (OR 0.608, 95% CI 0.362-1.021; *P* = 0.060). A similar pattern was observed for isolated CLNM versus no LNM (OR 0.594, 95% CI 0.346-1.017; P = 0.058). These findings indicate that the observed association was directionally consistent across several analyses but was attenuated in more restrictive and age-focused models, suggesting that residual confounding, particularly by age, cannot be fully excluded.

**Table 4:** Sensitivity analyses for the association between reproductive history and nodal metastasis.

Analysis method	Cohort	Outcome	Estimate 95% CI	*P* value
Crude logistic regression	Overall cohort (n = 1,828)	Any LNM	0.335 0.227-0.494	<0.001^a^
Adjusted logistic regression	Overall cohort (n = 1,828)	Any LNM	0.420 0.276-0.640	<0.001^a^
Logistic regression with alternative adjustment set	Overall cohort (n = 1,828)	Any LNM	0.417 0.273-0.638	<0.001^a^
Multivariable logistic regression	No LNM and isolated CLNM cohort(n=1497)	Isolated CLNM vs No LNM	0.405 0.260-0.630	<0.001^a^
Multivariable logistic regression	No LNM and isolated CLNM +LLNM cohort(n=1204)	CLNM+LLNM vs No LNM	0.391 0.220-0.696	0.001^a^
Restricted cohort analysis	Patients with pathological lateral neck evaluation(n=1256)	Lateral involvement	0.418 0.254-0.689	0.001^a^
Restricted-cohort analysis excluding tumors <1.0 cm	Restricted cohort Tumors ≥1.0 cm (n=911)	Any LNM	0.534 0.288-0.990	0.046^a^
1:1 PSM + conditional logistic regression	Matched cohort (n = 274)	Any LNM	0.516 0.289–0.922	0.026^a^
Logistic regression with continuous age modeled using restricted cubic splines	Overall cohort (n = 1,828)	Any LNM	0.608 0.362–1.021	0.060
Logistic regression with continuous age modeled using restricted cubic splines	No LNM and isolated CLNM cohort(n=1497)	Isolated CLNM vs No LNM	0.594 0.346–1.017	0.058

^a^ Statistically significant (*P*<0.05). Sensitivity analyses included crude logistic regression, fully adjusted logistic regression, an alternative adjustment model using baseline confounders, restriction to tumors≥1 cm, restriction to patients with pathological lateral neck evaluation, and 1:1 propensity score matching followed by conditional logistic regression. Propensity scores were estimated using age, BMI, hypertension, type 2 diabetes mellitus, history of malignancy, Hashimoto’s thyroiditis, TSH, tumor size, multifocality, bilaterality, and extrathyroidal extension. In the spline-adjusted models, the binary age term was replaced by restricted cubic spline terms for continuous age, while the remaining covariates were kept consistent with the primary adjusted model. Restricted cubic spline models used continuous age with four knots, replacing the binary age term used in the primary models. Effect estimates are reported as odds ratios with 95% confidence intervals. No adjustment for multiple comparisons across numerous models/outcomes.

### Category-specific associations of reproductive factors

**Figure 2:**
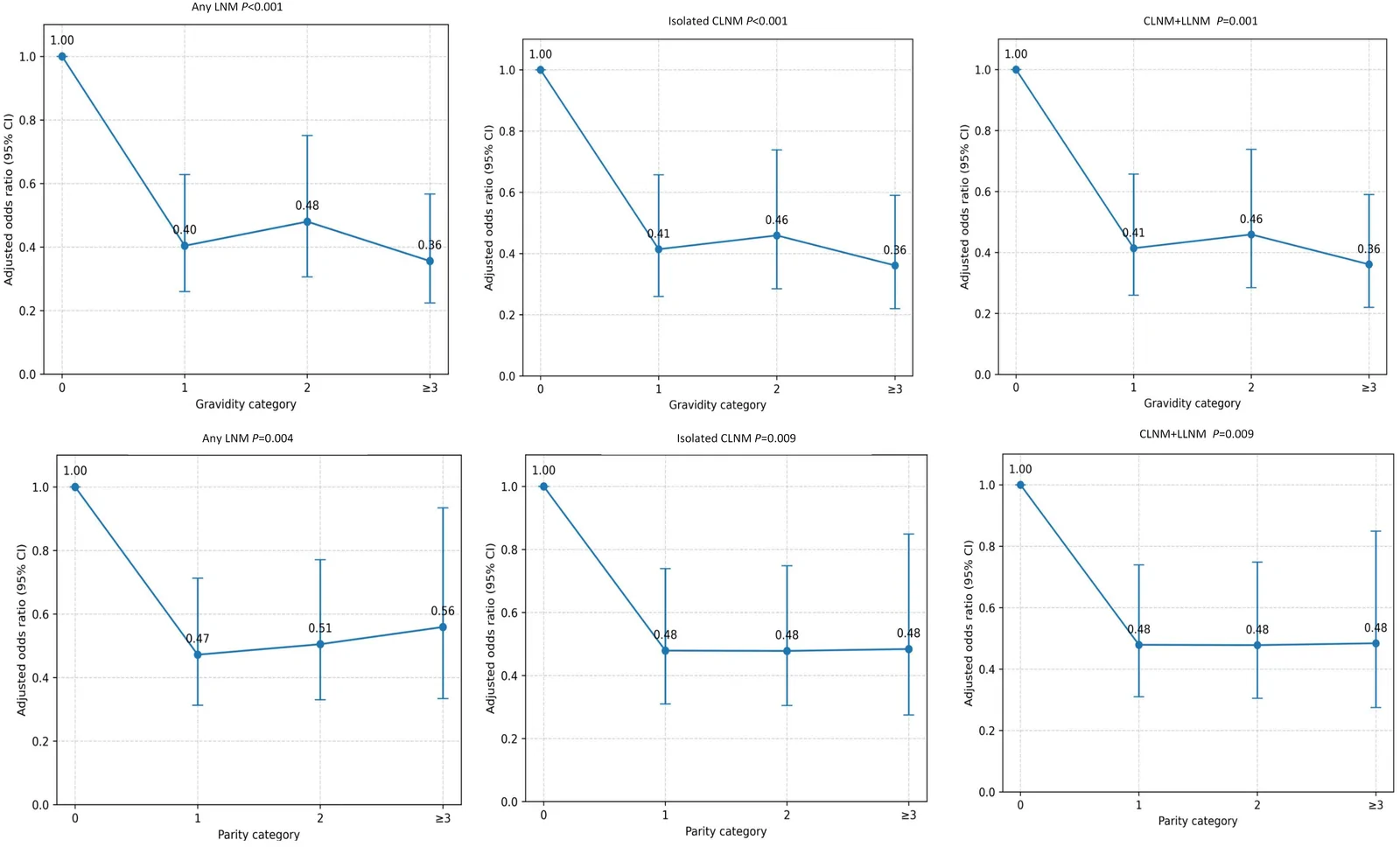
Category-specific associations of reproductive-history variables with nodal metastasis in women with papillary thyroid carcinoma. Adjusted odds ratios and 95% confidence intervals are shown for gravidity and parity categories across three nodal metastasis outcomes: any LNM, isolated CLNM, and combined CLNM+LLNM. Rows represent reproductive-history variables, and columns represent nodal metastasis outcomes. Patients without lymph node metastasis served as the reference group for all outcome models. Nulligravidity and nulliparity served as the reference categories for the corresponding reproductive-history variables. P for trend was calculated by modeling gravidity and parity as ordinal variables and should be interpreted cautiously because the category-specific estimates were not strictly monotonic. All models were adjusted for age, BMI, hypertension, type 2 diabetes mellitus, history of malignancy, Hashimoto’s thyroiditis, TSH, tumor size, multifocality, bilaterality, and extrathyroidal extension.

Category-specific analyses of gravidity, parity, and abortion history are shown in **Table 5** and **Figure 2**. Compared with nulligravid women, all gravidity categories were associated with lower odds of any LNM. The adjusted ORs were 0.404 (95% CI 0.260-0.628), 0.480 (95% CI 0.306-0.751), and 0.356 (95% CI 0.224-0.567) for women with 1, 2, and ≥3 pregnancies, respectively. Similar inverse associations were observed for isolated CLNM, with adjusted ORs of 0.414 (95% CI 0.260-0.657), 0.459 (95% CI 0.285-0.738), and 0.361 (95% CI 0.220-0.590) across increasing gravidity categories. For CLNM+LLNM, the association remained inverse, although effect estimates were less stable. However, the point estimates for gravidity were not strictly monotonic, suggesting that the pattern was more compatible with a threshold-like association between ever versus never pregnancy than with a linear dose-response relationship. Parity showed a similar threshold-like pattern. Although parous women had lower odds of Any LNM than nulliparous women, the inverse association did not become progressively stronger with increasing number of births. In the ever-gravid restricted analysis, abortion history was evaluated using women with gravidity but no abortion history as the reference group. Abortion history showed a more monotonic inverse pattern for Any LNM, but this association was mainly driven by the ≥2-abortion category, whereas the estimate for one abortion was not statistically significant. Therefore, abortion-history findings were interpreted as exploratory rather than as definitive evidence of an independent protective association.

**Table 5:** Dose-response associations of reproductive factors with nodal metastasis in women with PTC. (A). Any LNM VS. Reproductive-related factors (Gravidity Parity and Abortion history).

Panel A. Any LNM VS. Reproductive-related factors (Gravidity Parity and Abortion history)					
Outcome		Any LNM events (%)	Adjusted OR 95% CI	*P* value	*P* for trend
Gravidity	0	100(73%)	1.00 reference	—	<0.001^a^
	1	290(48.7%)	0.404 0.260-0.628	<0.001^a^	
	2	321(49.9%)	0.480 0.306-0.751	0.001^a^	
	≥3	192(42.6%)	0.356 0.224-0.567	<0.001^a^	
Parity	0	102(69.9%)	1.00 reference	—	0.004^a^
	1	375(47.5%)	0.472 0.313-0.713	<0.001^a^	
	2	335(47.7%)	0.505 0.330-0.771	0.002^a^	
	≥3	91(47.6%)	0.559 0.334-0.934	0.026^a^	
Abortion history	NO	629(49.4%)	1.00 reference		0.031^a^
	1	97(43.6%)	0.835 0.612-1.140	0.256	
	≥2	77(39.0%)	0.653 0.469-0.910	0.012^a^	
Panel B. Isolated CLNM VS. Reproductive-related factors (Gravidity Parity and Abortion history)					
Outcome		Isolated CLNM events (%)	Adjusted OR 95% CI	*P* value	*P* for trend
Gravidity	0	70(65.4%)	1.00 reference	—	0.001^a^
	1	189(38.2%)	0.414 0.260-0.657	<0.001^a^	
	2	191(37.2%)	0.459 0.285-0.738	0.001^a^	
	≥3	122(31.9%)	0.361 0.220-0.590	<0.001^a^	
Parity	0	72(62.1%)	1.00 reference	—	0.009^a^
	1	247(37.4%)	0.479 0.310-0.739	0.001^a^	
	2	205(35.8%)	0.478 0.305-0.748	0.001^a^	
	≥3	48(32.4%)	0.484 0.275-0.849	0.011^a^	
Abortion history	NO	388(37.6%)	1.00 reference		0.149
	1	56(30.9%)	0.756 0.529-1.081	0.125	
	≥2	58(32.5%)	0.775 0.543-1.104	0.158	
Panel C. CLNM+LLNM VS. Reproductive-related factors (Gravidity Parity and Abortion history)					
Outcome		CLNM+LLNM events (%)	Adjusted OR 95% CI	*P* value	*P* for trend
Gravidity	0	29(43.9%)	1.00 reference	—	0.002^a^
	1	87(22.1%)	0.298 0.154-0.580	<0.001^a^	
	2	105(24.6%)	0.403 0.207-0.783	0.007^a^	
	≥3	58(18.2%)	0.287 0.142-0.581	0.001^a^	
Parity	0	29(39.7%)	1.00 reference	—	0.005^a^
	1	108(20.7%)	0.362 0.192-0.681	0.002^a^	
	2	106(22.4%)	0.457 0.240-0.871	0.017^a^	
	≥3	36(26.5%)	0.644 0.299-1.389	0.262	
Abortion history	NO	203(23.9%)	1.00 reference	—	0.024^a^
	1	31(19.8%)	0.853 0.516-1.410	0.535	
	≥2	16(11.7%)	0.420 0.224-0.786	0.007^a^	

^a^ Statistically significant (*P*<0.05). All tests were two-sided. Panel A shows associations with any cervical lymph node metastasis, Panel B with isolated central lymph node metastasis, and Panel C with CLNM+LLNM. For Panels A-C, all comparisons were made against the “No LNM only” group. Gravidity, parity, and abortion history were analyzed in separate multivariable models to reduce collinearity. Models were adjusted for age, BMI, hypertension, type 2 diabetes mellitus, history of malignancy, Hashimoto’s thyroiditis, TSH, tumor size, multifocality, bilaterality, and extrathyroidal extension. For the abortion-history analysis, categories were defined as gravidity without abortion(NO), one abortion(1), and two or more abortions(≥2). The trend tests should be interpreted cautiously in light of the non-monotonic estimates.

### Prediction performance

**Figure 3:**
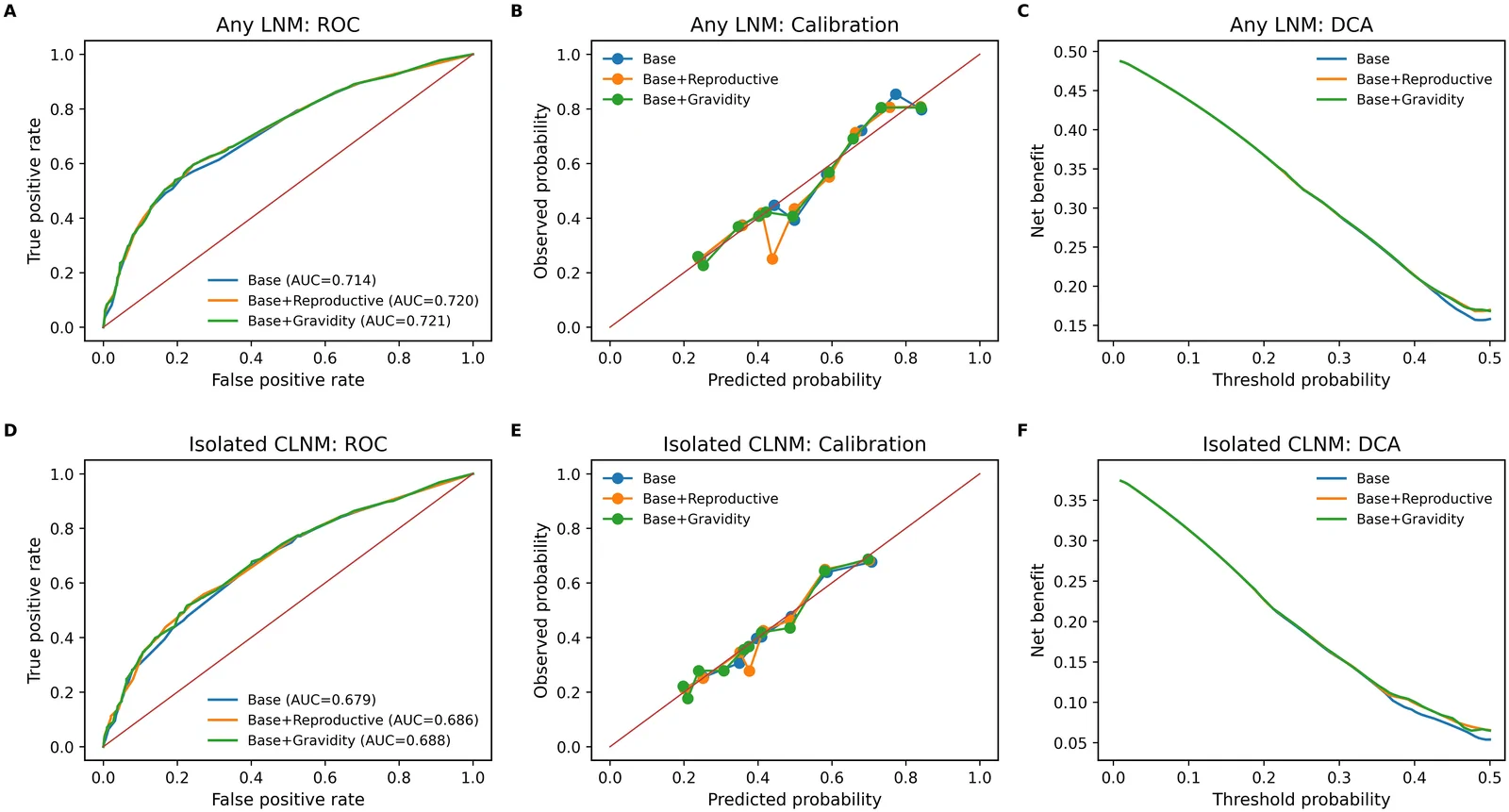
Predictive performance of clinicopathological models with and without reproductive factors. **(A–C)** show receiver operating characteristic curves **(A)**, calibration plots **(B)**, and decision curve analysis **(C)** for predicting any lymph node metastasis. **(D–F)** show the corresponding analyses for isolated central lymph node metastasis in a restricted cohort including only patients with isolated CLNM and those without lymph node metastasis. The baseline clinicopathological model was compared with models incorporating reproductive history or gravidity. The baseline clinicopathological, model was compared with models incorporating reproductive history or gravidity.

Prediction analyses showed that the baseline clinicopathological model had acceptable discrimination for any LNM. The addition of reproductive history or gravidity provided modest incremental improvements in model performance (**Figure 3**). Calibration remained adequate across models, and decision curve analysis suggested a small increase in net clinical benefit after inclusion of reproductive variables within clinically relevant threshold ranges. Similar patterns were observed in models restricted to isolated CLNM, although the magnitude of improvement was limited. Overall, these findings suggest that reproductive factors may contribute supplementary predictive information beyond conventional clinicopathological variables, but their incremental value was modest.

## Discussion

In this retrospective cohort of 1,828 women with papillary thyroid carcinoma, reproductive history was consistently associated with lower odds of cervical nodal metastasis in the primary adjusted models. This association was most evident for any LNM and isolated CLNM, whereas estimates for lateral nodal disease and CLNM+LLNM were directionally similar but less stable. Importantly, the association was attenuated after additional age-focused analyses, including flexible adjustment for continuous age using restricted cubic splines. Therefore, the present findings should be interpreted as an observational association rather than evidence of an independent protective effect of reproductive history.

These findings should be considered in the context of the broader PTC literature and our previous work. Lymph node metastasis remains a clinically important feature in PTC because it is associated with recurrence risk, surgical extent, and postoperative surveillance burden. Established clinicopathological correlates of nodal disease include age, tumor size, multifocality, bilaterality, extrathyroidal extension, and nodal compartment involvement (5–9). Previous studies have shown that younger patients with PTC are more likely to have lymph node metastasis and higher nodal burden (9, 19, 20). In our previous study, female sex was associated with a lower risk of nodal metastasis, and reproductive factors appeared to be related to nodal risk among women (14). The present female-only cohort extends that observation by suggesting that reproductive experience may capture part of the heterogeneity in nodal metastatic burden among women. However, because reproductive history is strongly correlated with age—a central potential confounder in our research—this signal should not be attributed to reproductive exposure alone.

Central and lateral nodal metastases should not be treated as interchangeable outcomes. Central lymph node metastasis is usually the earliest and most common pattern of regional spread in PTC, whereas lateral nodal disease often reflects a broader extent of dissemination and has more direct implications for operative planning (5, 15–18). In the present study, the inverse association between reproductive history and nodal disease was more stable for isolated CLNM than for lateral involvement. This may partly reflect biological differences between central and lateral nodal spread, but it may also reflect methodological factors. Pathological lateral neck evaluation was not performed uniformly across the cohort, and lateral nodal outcomes were more vulnerable to selection bias, occult disease, and sparse-event instability. Accordingly, findings involving lateral disease should be interpreted cautiously.

The sensitivity and prediction analyses further define both the strength and the limitations of the current evidence. The inverse association between reproductive history and any LNM was observed across several model specifications, but the estimates were attenuated in restricted, matched, and age-focused analyses. In particular, the patients with tumors ≥1.0 cm analysis and the propensity score-matched analysis yielded estimates close to the conventional significance boundary, and the isolated-CLNM association was attenuated after matching. In the stratified analysis of the primary models, although women with a history of pregnancy had lower odds of LNM than nulligravid women, the estimates did not decrease steadily with increasing gravidity. Similarly, the parity results appeared to reflect a contrast between parous and nulliparous women rather than a progressively lower risk with additional births. Prediction analyses suggest that adding reproductive history or gravidity to clinicopathological models provided only modest incremental improvement. The abortion-history analysis was also not consistent across all nodal endpoints and was mainly driven by the ≥2-abortion category. Thus, reproductive variables may provide supplementary descriptive or predictive information, but their added value appears limited and should be considered exploratory.

The literature on reproductive factors and thyroid cancer has been inconsistent, particularly when the endpoint is cancer incidence rather than metastatic phenotype. Some epidemiologic studies and meta-analyses have suggested associations between menstrual or reproductive variables and thyroid cancer risk, whereas others have reported weak, null, or directionally mixed findings (10–13, 21). Similarly, studies evaluating pregnancy during active surveillance of papillary thyroid microcarcinoma have reported heterogeneous observations, including occasional tumor enlargement but limited evidence of clinically meaningful nodal progression during gestation (22, 23). Taken together, these data suggest that reproductive hormonal exposure may be related to thyroid tumor biology, but not in a simple or uniform way. Our findings add to this literature by examining nodal metastatic patterns among women with established PTC rather than thyroid cancer incidence alone.

The biological explanation remains uncertain, and causal inference cannot be made from the present study. Pregnancy and lactation are accompanied by complex, time-dependent endocrine, immune, inflammatory, and metabolic changes. However, the present dataset did not include circulating reproductive hormones, tumor hormone-receptor status, lactation-related markers, age at first pregnancy, breastfeeding history, menopausal status, or socioeconomic factors. Therefore, the observed inverse associations may reflect unmeasured endocrine, reproductive, clinical, or socioeconomic factors, residual confounding, or selection effects rather than a direct effect of reproductive history per se. Prior experimental and clinicopathological studies indicate that estrogen-related signaling, including ER-dependent pathways and ERK activation, is involved in thyroid tumor biology (24–26). Some studies have linked ERα or progesterone receptor expression with proliferative or more aggressive features, whereas ERβ-related effects may differ by isoform, subcellular localization, and tumor context (26, 27). These data highlight the biological complexity of sex-hormone signaling in thyroid cancer and do not support a simple interpretation that the high-estrogen state of pregnancy protects against nodal metastasis. The mechanistic interpretation of our findings should therefore be considered hypothesis-generating only.

Overall, the present study suggests that ever pregnancy is associated with lower odds of cervical nodal metastasis in women with PTC in primary adjusted analyses, particularly for central compartment disease. However, this association was attenuated after additional age-focused analyses, and the category-specific gravidity and parity patterns did not support a strict dose-response relationship. The most appropriate interpretation is that reproductive history may be associated with nodal metastatic burden as part of a broader host-related context, rather than acting as an independent protective factor. Future prospective studies incorporating reproductive endocrine markers, more detailed reproductive and socioeconomic variables, standardized nodal assessment, and long-term oncologic outcomes are needed to clarify these associations and to determine whether reproductive information can improve clinically applicable risk prediction models. From a clinical standpoint, these findings should not be used to alter the scope of surgery based solely on reproductive history. Surgical decision-making in PTC should continue to be guided primarily by age, tumor size, multifocality, bilaterality, extrathyroidal extension, imaging findings, cytology or pathology, and compartment-oriented nodal assessment. Reproductive history may provide additional clinical context in selected female patients, particularly when the preoperative risk profile is otherwise borderline or heterogeneous, but it should not independently determine the extent of central or lateral neck dissection.

This study has several strengths. It included a relatively large female - only surgical cohort, used pathology - based nodal outcomes, evaluated multiple reproductive history dimensions, and incorporated sensitivity, subgroup and prediction analyses. Several limitations should also be acknowledged. First, the study was retrospective and single - center, and the cohort was drawn from a Chinese population during 2015–2019, a period in which reproductive patterns may have been influenced by family-planning policies; therefore, generalizability may be limited. Second, reproductive variables were derived from medical records and patient-reported history and may have been misclassified. Third, although women with missing reproductive-history data were excluded before construction of the final analytic cohort, detailed reproductive variables such as age at first pregnancy, breastfeeding history, menopausal status, and socioeconomic factors were not available. Fourth, analyses involving lateral nodal disease were susceptible to selection bias because pathological lateral neck evaluation was not uniform. Fifth, sparse events limited the precision of some subgroup and pattern - specific estimates. Sixth, residual confounding by age remains an important concern, as nulligravid women were substantially younger than ever-gravid women and spline-adjusted analyses attenuated the reproductive-history estimates. Finally, mechanistic endocrine markers and long - term oncologic outcomes were not available in the finalized analytic dataset, limiting biological interpretation and prognostic inference.

## Conclusion

In this female surgical cohort of papillary thyroid carcinoma, reproductive history was associated with a lower likelihood of cervical lymph node metastasis, particularly central compartment disease. This association appeared to be more closely related to ever having been pregnant than to a linear increase in gravidity or parity. Reproductive information may provide supplementary clinical context for nodal risk assessment, but it should not replace established clinicopathological factors or independently alter surgical decision-making. Further prospective studies incorporating detailed reproductive, endocrine, and long-term oncologic data are needed to clarify these associations.

## Data Availability

The original contributions presented in the study are included in the article/**Supplementary Material**. Further inquiries can be directed to the corresponding author.

## Supplementary material

Supplementary Table 1 Full multivariable logistic regression models including all candidate covariates for pathological nodal metastatic patterns

Supplementary Table 2 The age distribution by gravidity category. This shows that nulligravid women were substantially younger than ever-gravid women.

Supplementary Table 3 LNM vs Gravidity within age strata This demonstrates that the reproductive association persists within age strata or in an age-restricted analysis.

## References

[1] Bray F Laversanne M Sung H Ferlay J Siegel RL Soerjomataram I . Global cancer statistics 2022: GLOBOCAN estimates of incidence and mortality worldwide for 36 cancers in 185 countries. CA Cancer J Clin. (2024) 74:229–63. doi: 10.3322/caac.21834 38572751

[2] Davies L Hoang JK . Thyroid cancer in the USA: current trends and outstanding questions. Lancet Diabetes Endocrinol. (2021) 9:11–2. doi: 10.1016/s2213-8587(20)30372-7 33220765

[3] Cui Y Mubarik S Li R Nawsherwan Yu C . Trend dynamics of thyroid cancer incidence among China and the U.S. adult population from 1990 to 2017: a joinpoint and age-period-cohort analysis. BMC Public Health. (2021) 21:624. doi: 10.1186/s12889-021-10635-w 33789605 PMC8010947

[4] Lim H Devesa SS Sosa JA Check D Kitahara CM . Trends in thyroid cancer incidence and mortality in the United States, 1974-2013. JAMA. (2017) 317:1338–48. doi: 10.1001/jama.2017.2719 28362912 PMC8216772

[5] Haugen BR Alexander EK Bible KC Doherty GM Mandel SJ Nikiforov YE . 2015 American Thyroid Association Management Guidelines for adult patients with thyroid nodules and differentiated thyroid cancer. Thyroid. (2016) 26:1–133. doi: 10.1089/thy.2015.0020 26462967 PMC4739132

[6] Giordano D Valcavi R Thompson GB Pedroni C Renna L Gradoni P . Complications of central neck dissection in patients with papillary thyroid carcinoma: results of a study on 1087 patients and review of the literature. Thyroid. (2012) 22:911–7. doi: 10.1089/thy.2012-0011 22827494

[7] Kim SY Kim BW Pyo JY Hong SW Chang HS Park CS . Macrometastasis in papillary thyroid cancer patients is associated with higher recurrence in lateral neck nodes. World J Surg. (2018) 42:123–9. doi: 10.1007/s00268-017-4158-5 28779384

[8] Lee YM Sung TY Kim WB Chung KW Yoon JH Hong SJ . Risk factors for recurrence in patients with papillary thyroid carcinoma undergoing modified radical neck dissection. Br J Surg. (2016) 103:1020–5. doi: 10.1002/bjs.10144 27121346

[9] Mao J Zhang Q Zhang H Zheng K Wang R Wang G . Risk factors for lymph node metastasis in papillary thyroid carcinoma: a systematic review and meta-analysis. Front Endocrinol (Lausanne). (2020) 11:265. doi: 10.3389/fendo.2020.00265 32477264 PMC7242632

[10] Caini S Gibelli B Palli D Saieva C Ruscica M Gandini S . Menstrual and reproductive history and use of exogenous sex hormones and risk of thyroid cancer among women: a meta-analysis of prospective studies. Cancer Causes Control. (2015) 26:511–8. doi: 10.1007/s10552-015-0546-z 25754110

[11] Cao Y Wang Z Gu J Hu F Qi Y Yin Q . Reproductive factors but not hormonal factors associated with thyroid cancer risk: a systematic review and meta-analysis. BioMed Res Int. (2015) 2015:103515. doi: 10.1155/2015/103515 26339585 PMC4538312

[12] Rossing MA Voigt LF Wicklund KG Daling JR . Reproductive factors and risk of papillary thyroid cancer in women. Am J Epidemiol. (2000) 151:765–72. doi: 10.1093/oxfordjournals.aje.a010276 10965973

[13] Schonfeld SJ Ron E Kitahara CM Brenner A Park Y Sigurdson AJ . Hormonal and reproductive factors and risk of postmenopausal thyroid cancer in the NIH-AARP Diet and Health Study. Cancer Epidemiol. (2011) 35:e85–90. doi: 10.1016/j.canep.2011.05.009 21852218 PMC3215902

[14] Shi P Yang D Liu Y Zhao Z Song J Shi H . A protective factor against lymph node metastasis of papillary thyroid cancer: female gender. Auris Nasus Larynx. (2023) 50:440–9. doi: 10.1016/j.anl.2022.10.001 36253315

[15] Cai H Zhuge L Huang Z Wang S Shi P Yan D . Distinct risk factors of lateral lymph node metastasis in patients with papillary thyroid cancer based on age stratification. BMC Surg. (2024) 24:24. doi: 10.1186/s12893-024-02309-2 38218911 PMC10787958

[16] Cai H Zhuge L Huang Z Wang S Shi P Yan D . Predictive value of jugulo-omohyoid lymph nodes in lateral lymph node metastasis of papillary thyroid cancer. BMC Endocr Disord. (2024) 24:74. doi: 10.1186/s12902-024-01576-7 38773428 PMC11106992

[17] Lonjon G Porcher R Ergina P Fouet M Boutron I . Potential pitfalls of reporting and bias in observational studies with propensity score analysis assessing a surgical procedure: a methodological systematic review. Ann Surg. (2017) 265:901–9. doi: 10.1097/sla.0000000000001797 27232253

[18] Song M Huang Z Wang S Huang J Shi H Liu Y . Predictive factors of lateral lymph node metastasis in conventional papillary thyroid carcinoma. Gland Surg. (2020) 9:1000–7. doi: 10.21037/gs-20-482 32953608 PMC7475366

[19] Shukla N Osazuwa-Peters N Megwalu UC . Association between age and nodal metastasis in papillary thyroid carcinoma. Otolaryngol Head Neck Surg. (2021) 165:43–9. doi: 10.1177/0194599820966995 33076796

[20] Wang W Ding Y Meng C Li P Bai N Li X . Patient's age with papillary thyroid cancer: Is it a key factor for cervical lymph node metastasis? Eur J Surg Oncol. (2023) 49:1147–53. doi: 10.1016/j.ejso.2023.02.011 36863913

[21] Braganza MZ Berrington de González A Schonfeld SJ Wentzensen N Brenner AV Kitahara CM . Benign breast and gynecologic conditions, reproductive and hormonal factors, and risk of thyroid cancer. Cancer Prev Res (Phila). (2014) 7:418–25. doi: 10.1158/1940-6207.capr-13-0367 24449056 PMC3976437

[22] Shindo H Amino N Ito Y Kihara M Kobayashi K Miya A . Papillary thyroid microcarcinoma might progress during pregnancy. Thyroid. (2014) 24:840–4. doi: 10.1089/thy.2013.0527 24397849

[23] Ito Y Miyauchi A Kudo T Ota H Yoshioka K Oda H . Effects of pregnancy on papillary microcarcinomas of the thyroid re-evaluated in the entire patient series at Kuma Hospital. Thyroid. (2016) 26:156–60. doi: 10.1089/thy.2015.0393 26670937 PMC4739387

[24] Zeng Q Chen GG Vlantis AC van Hasselt CA . Oestrogen mediates the growth of human thyroid carcinoma cells via an oestrogen receptor-ERK pathway. Cell Prolif. (2007) 40:921–35. doi: 10.1111/j.1365-2184.2007.00471.x 18021179 PMC6495898

[25] Gong Z Yang S Wei M Vlantis AC Chan JYK van Hasselt CA . The isoforms of estrogen receptor alpha and beta in thyroid cancer. Front Oncol. (2022) 12:916804. doi: 10.3389/fonc.2022.916804 35814443 PMC9263191

[26] Huang Y Dong W Li J Zhang H Shan Z Teng W . Differential expression patterns and clinical significance of estrogen receptor-α and β in papillary thyroid carcinoma. BMC Cancer. (2014) 14:383. doi: 10.1186/1471-2407-14-383 24884830 PMC4049482

[27] Vannucchi G De Leo S Perrino M Rossi S Tosi D Cirello V . Impact of estrogen and progesterone receptor expression on the clinical and molecular features of papillary thyroid cancer. Eur J Endocrinol. (2015) 173:29–36. doi: 10.1530/eje-15-0054 25862786

