# Supplementary material for: Association of gravidity, parity, and abortion history with nodal metastasis in women with papillary thyroid carcinoma: a retrospective cohort study
Source: Front Endocrinol (Lausanne). 2026 Aug 6;17:1856799. doi: 10.3389/fendo.2026.1856799 (PMC13489909; doi:10.3389/fendo.2026.1856799)
Supplement: Supplementary file 1 [file SupplementaryFile1.docx]

Supplementary Table 1. Full multivariable logistic regression models including all candidate covariates for pathological nodal metastatic patterns

| **Variable** | **Any LNM vs No LNM OR 95% CI** | ***P* value** | **Isolated CLNM vs No LNM OR 95% CI** | ***P* value** | **Isolated LLNM vs No LNM OR 95% CI** | ***P* value** | **Lateral involvement vs No LNM OR 95% CI** | ***P* value** | **CLNM+LLNM vs No LNM OR 95% CI** | ***P* value** |
| --- | --- | --- | --- | --- | --- | --- | --- | --- | --- | --- |
| Reproductive history (yes vs no) | 0.417 0.273-0.638 | <0.001^a^ | 0.415 0.266-0.648 | <0.001^a^ | 1.727 0.220-13.585 | 0.603 | 0.400 0.219-0.731 | 0.003^a^ | 0.331 0.176-0.623 | 0.001^a^ |
| Age, years | 0.452 0.362-0.565 | <0.001^a^ | 0.432 0.338-0.554 | <0.001^a^ | 1.017 0.540-1.918 | 0.957 | 0.516 0.372-0.714 | <0.001^a^ | 0.426 0.297-0.611 | <0.001^a^ |
| Hashimoto’s thyroiditis | 1.062 0.737-1.531 | 0.746 | 0.808 0.529-1.233 | 0.323 | 2.544 1.158-5.588 | 0.020^a^ | 1.649 0.997-2.728 | 0.051 | 1.353 0.759-2.410 | 0.305 |
| Tumor size >1 cm | 3.038 2.441-3.781 | <0.001^a^ | 2.396 1.877-3.057 | <0.001^a^ | 2.130 1.153-3.935 | 0.016^a^ | 4.935 3.657-6.659 | <0.001^a^ | 5.976 4.312-8.283 | <0.001^a^ |
| Multifocality | 1.291 0.933-1.784 | 0.123 | 1.078 0.748-1.554 | 0.687 | 1.337 0.576-3.105 | 0.500 | 1.762 1.110-2.796 | 0.016^a^ | 1.791 1.067-3.005 | 0.027^a^ |
| Bilaterality | 1.583 1.095-2.289 | 0.015^a^ | 1.412 0.926-2.152 | 0.109 | 0.881 0.323-2.404 | 0.804 | 1.906 1.158-3.135 | 0.011^a^ | 2.300 1.328-3.983 | 0.003^a^ |
| Extrathyroidal extension | 1.929 1.484-2.508 | <0.001^a^ | 1.605 1.193-2.159 | 0.002^a^ | 2.332 1.203-4.521 | 0.012^a^ | 2.629 1.870-3.695 | <0.001^a^ | 2.783 1.929-4.014 | <0.001^a^ |
| BMI >25 kg/m² | 0.933 0.758-1.148 | 0.512 | 0.951 0.755-1.199 | 0.671 | 0.960 0.537-1.717 | 0.891 | 0.844 0.625-1.140 | 0.269 | 0.830 0.596-1.154 | 0.267 |
| Hypertension | 1.191 0.896-1.584 | 0.228 | 1.176 0.850-1.627 | 0.327 | 0.692 0.301-1.588 | 0.385 | 1.153 0.775-1.716 | 0.482 | 1.357 0.880-2.092 | 0.167 |
| Type 2 diabetes mellitus | 0.701 0.449-1.093 | 0.117 | 0.746 0.448-1.242 | 0.260 | 1.157 0.383-3.494 | 0.797 | 0.717 0.377-1.363 | 0.310 | 0.670 0.324-1.387 | 0.281 |
| History of malignancy | 0.831 0.469-1.474 | 0.526 | 1.014 0.552-1.864 | 0.964 | 0.450 0.058-3.502 | 0.446 | 0.462 0.172-1.239 | 0.125 | 0.446 0.150-1.325 | 0.146 |
| TSH, mIU/L | 0.971 0.922-1.022 | 0.254 | 0.982 0.778-1.239 | 0.880 | 0.911 0.501-1.655 | 0.759 | 0.843 0.621-1.143 | 0.271 | 0.805 0.576-1.125 | 0.203 |

Supplementary Table 2. The age distribution by gravidity category.

| **Gravidity** | Age,years | Median age,years |
| --- | --- | --- |
| 0 | 18-52 | 27.80±6.02 |
| 1 | 23-70 | 43.65±10.31 |
| 2 | 22-77 | 47.84±10.87 |
| ≥3 | 26-78 | 50.08±10.81 |

This shows that nulligravid women were substantially younger than ever-gravid women.

Supplementary Table 3.LNM vs Gravidity within age strata

| Age,years | Any LNM **OR 95% CI** | *P* value |
| --- | --- | --- |
| <30 | 0.512(0.246-1.065) | 0.073 |
| 30-45 | 0.503(0.226-1.121) | 0.093 |
| 46-60 | 0.417(0.270-0.644) | <0.001 |
| ≥61 | All patients in this group had a documented reproductive history. | |

This demonstrates that the reproductive association persists within age strata or in an age-restricted analysis.
